# Novel Anatomical Proposal for Botulinum Neurotoxin Injection Targeting Lateral Canthal Rhytids

**DOI:** 10.3390/toxins14070462

**Published:** 2022-07-06

**Authors:** Kyu-Ho Yi, Ji-Hyun Lee, Ga-Young Kim, Seong-Wook Yoon, Wook Oh, Hee-Jin Kim

**Affiliations:** 1 Wonju Public Health Center, Wonjusi 26417, Korea; kyuho90@daum.net; 2 Division in Anatomy and Developmental Biology, Department of Oral Biology, Human Identification Research Institute, BK21 PLUS Project, Yonsei University College of Dentistry, 50-1 Yonsei-ro, Seoul 03722, Korea; jhlee119@yuhs.ac; 3 Institute of Biomedical Communications, Incheon Catholic University, Incheon 23058, Korea; babycat828@gmail.com; 4 BongHwa Public Health Center, Bonghwa-gun 36238, Korea; seongwook94@gmail.com; 5 Maylin Clinic (Yeouido), Seoul 07335, Korea; feelclinic@naver.com

**Keywords:** orbicularis oculi muscle, infraorbital wrinkles, facial wrinkle, injection point, crow’s feet

## Abstract

Botulinum neurotoxin injections near the lateral canthal rhytids are commonly used in cosmetic settings; however, there is a lack of thorough anatomical knowledge, and an effective way to treat them with accumulating knowledge is needed. The anatomical characteristics concerning the injection of botulinum neurotoxin into the orbicularis oculi muscle were evaluated in this review. Current knowledge on the identification of botulinum neurotoxin injection points from recent anatomical research was assessed. The lateral canthal lines are involved with the orbicularis oculi muscle and nearby anatomical structures, and the injection points can be more precisely defined. The best possible injection sites were provided, and the injection procedure was described. This review proposes evidence for injection sites associated with the surface anatomy of the orbicularis oculi muscles to enhance the effectiveness of easing lateral canthal rhytids.

## 1. Introduction

**Figure 1:**
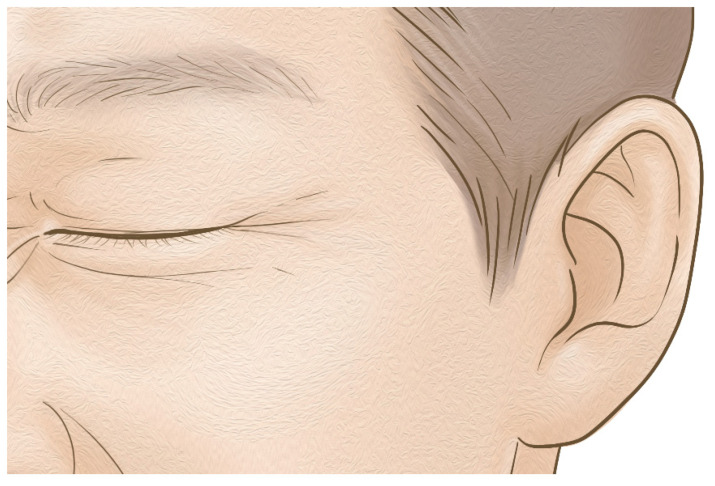
The wrinkles in the eye region are lateral canthal rhytids in many individuals.

Botulinum neurotoxin (BoNT) stops neuronic linking by seizing the release of acetylcholine at the neuromuscular junction and hindering contraction of the muscle [1,2]. In cosmetic treatment, BoNT is usually used to reduce periorbital wrinkles by diminishing expression in the orbicularis oculi muscle. The main aesthetic concerns in the periorbital region are lateral canthal rhytids (crow’s feet) (Figure 1).

The easing of wrinkles through BoNT is performed in numerous cases, and side effects, such as paralysis of nearby muscles causing diplopia and ptosis, have been reported [3,4]. In treating creases with BoNT in the periorbital region, major complications such as diplopia may be caused by inadvertent palsy of the rectus lateralis, medialis, and inferioris [5,6]. Even if there is a low incidence of adverse effects, clinicians should inject anatomically-based exact sites of the orbicularis oculi muscle, and primary treatment should be initiated with a reduced amount of BoNT.

**Figure 2:**
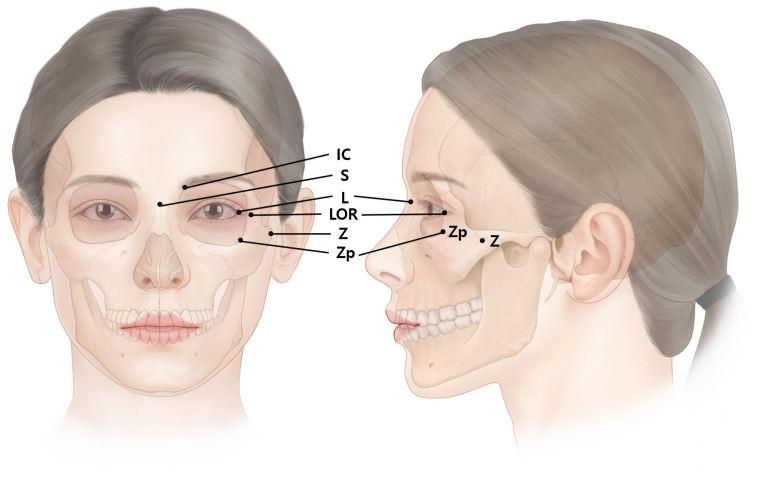
The external anatomical landmark of the eye regions. LOR, lateral orbital rim at the level of lateral canthus; S, sellion; Z, zygion; Zp, zygomatic point located on the outer orbital region; L, lateral canthus; IC, interciliary point located on the frontal notch.

Factors that should be considered are that a substantial number of units and short-term repetitive injections of BoNT build up antibodies that lead to insufficient treatment [7,8,9,10]. Recent findings on BoNT injection points from anatomical perspectives in certain muscles have been based on external anatomical landmarks (Figure 2) [11,12]. This review aimed to propose safe and efficient BoNT injection points and techniques for lateral canthal lines.

## 2. Anatomy of the Orbicularis Oculi Muscle

**Figure 3:**
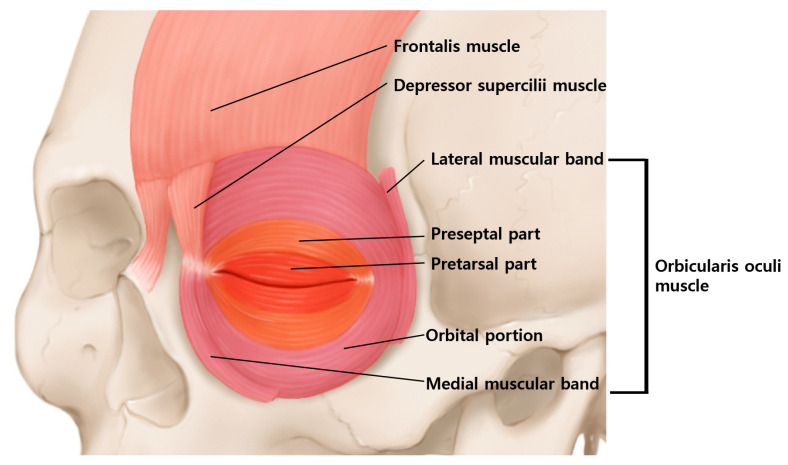
Schematic image of the orbicularis oculi muscle. The orbicularis oculi muscle is divided into the orbital portion and the palpebral portion according to their position. The palpebral portion is subdivided into preseptal and pretarsal portions.

The orbicularis oculi muscle is located around the eyes, and it acts to close the eyelid. The orbicularis oculi muscle can be divided into four parts: orbital portion, palpebral portion, lateral band, and medial band of the orbicularis oculi muscle. The palpebral portion can be subdivided into preseptal and pretarsal portions according to their location (Figure 3).

The orbital portion is in the outer region and the muscle originates from both the supraorbital and infraorbital margins. The muscle then encircles and inserts into the lateral canthal tendon, medial canthal tendon, partially to the frontalis muscle, procerus muscle, corrugator supercilii muscles and skin.

**Figure 4:**
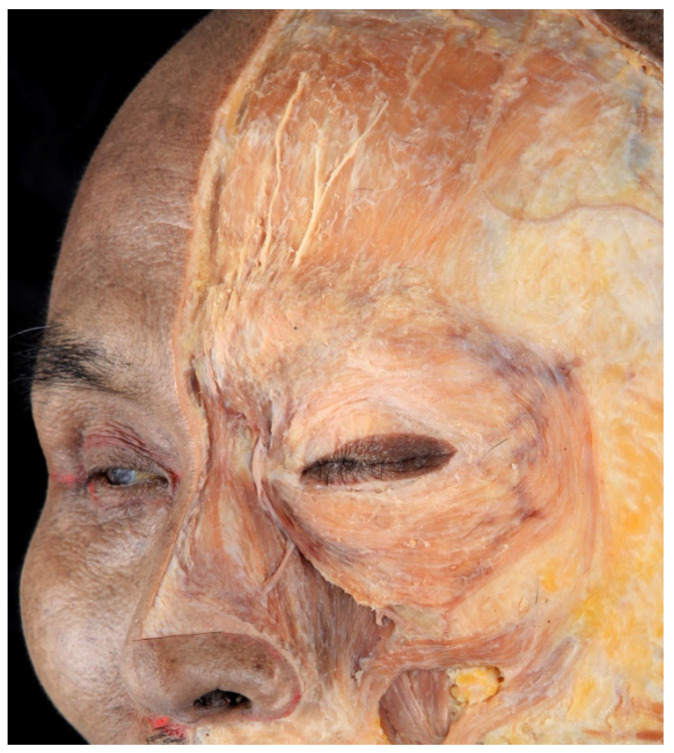
Dissected image of orbicularis orculi muscle. The muscle is composed of lateral and medial muscular band, preseptal and pretarsal part, and orbital portion.

In a study by Kim et al., the mean distance from the lateral canthus to the lateral edge of the orbicularis oculi muscle was 31 mm. The medial band of the orbicularis oculi muscle was observed in 64% of the population, whereas the lateral band was observed in 54% (Figure 4) [13].

According to Park et al., the lateral muscular band originating from the superficial temporal fascia lateral to the orbicularis oculi muscle was observed in half of the population [14]. The termination of the band was at the zygomatic arch region in 27.9%, at the cheek region 8%, and around mouth corner was 8.2%. This suggests that the lateral band may play a significant role in facial animation or cheek dimple formation and should be considered when treating lateral canthal rhytids.

The orbital portion of the orbicularis oculi muscle firmly closes the eyelids and pulls down the eyebrows. When a person smiles, this portion contracts and lateral canthal rhytids occur around the lateral corners of the eyes.

The palpebral portion consists of the preseptal and pretarsal parts. The preseptal part is located superficially to the orbital septum, whereas the pretarsal part is from the tarsal plate. The palpebral portion of the muscle acts voluntarily and involuntarily, shutting the eyelids when blinking. The palpebral portion produces vertical wrinkles in the medial canthus. The complicated muscle fibers of the orbicularis oculi muscle generate wrinkles in different directions, depending on the location.

**Figure 5:**
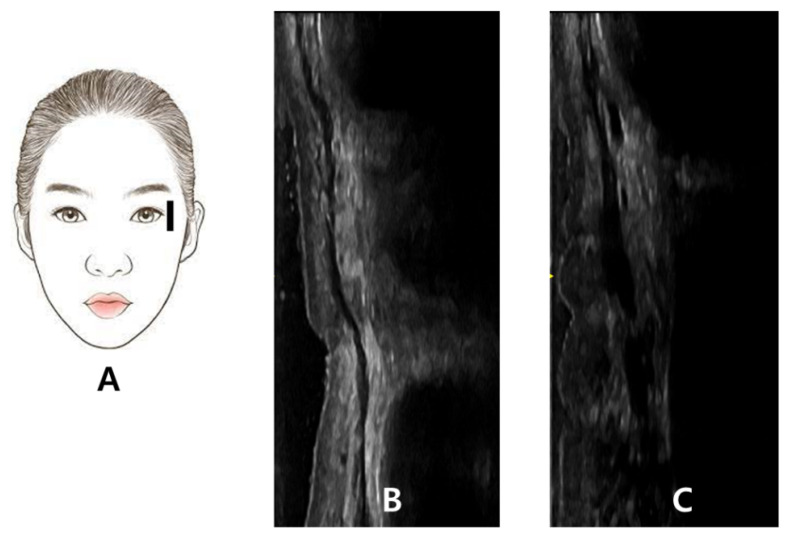
The probe of the ultrasonography has been placed longitudinally at lateral orbital rim at the level of lateral canthus (**A**). The thin hypoechoic orbicularis oculi muscle observed in ultrasonography during resting state (**B**). The thick hypoechoic orbicularis oculi muscle is observed in contracted state when asked to smile (**C**).

According to ultrasonographic findings, the lateral part of the orbicularis oculi muscle is the thickest and appears hypoechoic. The hypoechoic orbicularis oculi muscle continues superiorly with the hyperechoic temporoparietal fascia (Figure 5).

## 3. Injection Points for Lateral Canthal Rhytids

**Figure 6:**
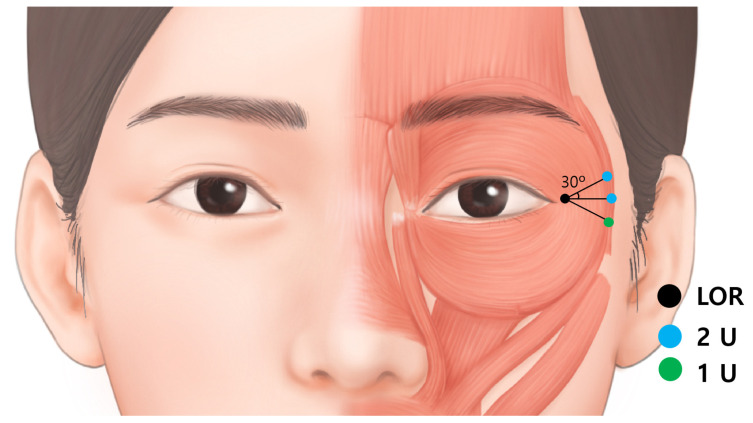
The injection points for lateral canthal rhytids (Crow’s feet). The light blue dot represents dose of 2 U, while green dot represents dose of 1 U. The degree of the superior and inferior injection is by 30 degrees. The total is 5 U, which is a satisfactory dose for each side. LOR, lateral orbital rim at the level of lateral canthus.

The anatomical landmark for lateral canthal rhytids is the lateral orbital rim at the level of the lateral canthus (Figure 6). From a point 20 mm apart in three directions, superior 30 degree, lateral and inferior 30 degree injection points are proposed.

Considering the extension of the orbicularis oculi muscle from the lateral canthus to the lateral edge of the orbicularis oculi muscle is around 3 cm [13] and the fact that BoNT spreads over 1 cm from the injection point, injection into a minimum of three points with 1–2 U of BoNT each, totaling 5 U, is recommended. Borodic et al. demonstrated that BoNT diffuses up to 4.5 cm at a point of injection in case of 10 U into the rabbit longissimus dorsi muscle and the 1 U injection diffusion gradient of 1.5 cm to 3 cm [15].

Next, 2 U were injected at each superior and lateral injection points; and 1 U was injected at the inferior injection point. Inferior point is proposed with lesser dose because, the diffusion of BoNT would affect zygomaticus major and minor giving asymmetrical facial expression. The total was 5 U on each side. The injection points were located around the border of the orbicularis oculi muscle. This is to avoid the venous damage of the sentinel and inferior palpebral veins that runs near the orbital rim. In addition, serious side effects such as diplopia can be prevented by injection away from the orbital rim. Although the injection is conducted in the margin of the orbicularis oculi muscle, BoNT diffuses to 1.5-3 cm which is sufficient to be effective in lateral canthal rhytids [16].

## 4. Depth

Subdermal or intradermal injection is recommended. As the orbicularis oculi muscle is in a layer extending to the superficial musculoaponeurotic system (SMAS) and temporoparietal fascia, superficial injection should be adequate.

Another factor to consider regarding the depth is the venous system. A bruise is the most common adverse effect of BoNT injection into lateral canthal rhytids. The venous system around the periorbital area is prone to bruising. In particular, the medial zygomaticotemporal vein (sentinel vein) and the inferior palpebral vein (Figure 6), should be carefully avoided.

## 5. Medial Zygomaticotemporal Vein

The medial zygomaticotemporal vein receives blood from the superior and lateral orbital rim (branches in the forehead, parietal and temporal areas) and drains into the middle temporal vein, running in the middle of the superficial and deep layers of the deep temporal fascia, and eventually becomes the superficial temporal vein [17]. In addition, the medial zygomaticotemporal vein branches inferiorly to the inferior palpebral vein. According to Yang et al., the piercing point of the medial zygomaticotemporal vein was located 26.8 ± 5.9 mm from the lateral canthus. Therefore, the injection points are duplicated with the range of the vein.

## 6. Inferior Palpebral Vein

**Figure 7:**
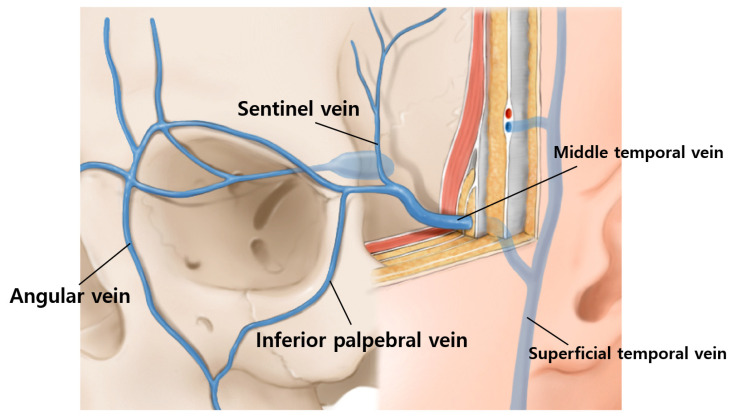
The veins of periorbital regions should be delicately avoided with superficial injection sentinel vein (medial zygomaticotemporal vein) and inferior palpebral vein.

The inferior palpebral vein receives blood from the inferior palpebral and lateral parts of the orbicularis oculi muscle and connects to the angular vein (Figure 7). Since the inferior palpebral vein is laterally connected to the medial zygomaticotemporal vein, the structure should be avoided before the injection. Subdermal or intradermal injections are ideal injection techniques to prevent bruising of the vein.

## 7. Discussion

To ease or prevent lateral canthal rhytids, which are also known as crow’s feet, the injection of BoNT into the lateral orbital portion is well known. However, anatomical considerations have not been well described even though this has led to side effects. Targeting lateral canthal rhytids with BoNT can cause major problems, such as diplopia, which may result from unintended blocking of the rectus inferioris or lateralis [5,6,12]. Although BoNT causing diplopia is uncommon, it can be critical to individuals [3,6]. Chen et al. reported a patient with BoNT in the lateral canthal region that caused lateral rectus paresis [5]. BoNT diffusion does not often affect nearby muscles, such as extraocular muscles, when treating lateral canthal rhytids. Supposedly, the extraocular muscles are separated from the injection sites but in certain circumstances, BoNT diffuses in the direction of the lateral rectus muscle. In these cases, diplopia typically occurs on one side; when it occurs, the medial rectus becomes hyperactive, causing the pupil to shift to the medial side.

Injecting BoNT into the superolateral portion of the eye regions may cause side effects of ptosis, which have been reported [18,19]. Eyelid ptosis can occur when a large dose of the injection is given, or when the injection site is close to the orbital rim. Eyelid ptosis occurs in the paralysis of levator palpebrae superioris muscle [20]. Although sensitivity to BoNT differs among individuals, there is no effective treatment for ptosis, which persists for several months [18,19]. Xerohphthalmia may occur because BoNT affects the palpebral portion of the orbicularis oculi muscle. The palpebral portion of the orbicularis oculi muscle may reduce involuntary blinking. In addition, xerophthalmia can be induced when BoNT is injected deep in the superolateral portion where the lacrimal glands are located beneath the orbicularis oculi muscle, and lacrimal secretion is decreased [20].

**Figure 8:**
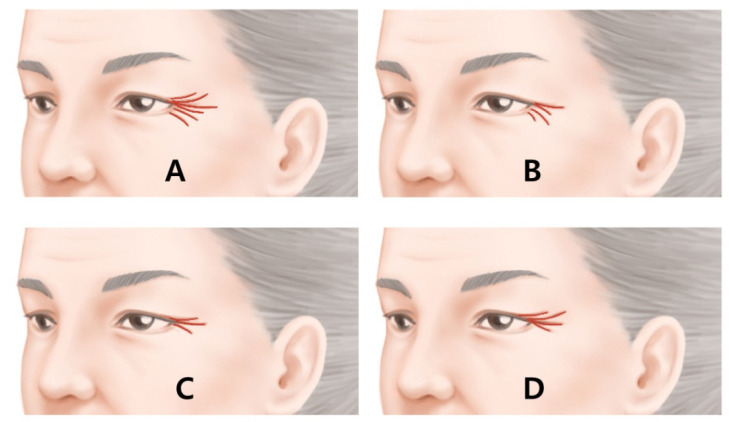
Four types of lateral canthal rhytids classified by kane. (**A**) full type, full dispersal of rhytid; (**B**) lower type, rhytid predominantly on the lower side of the lateral canthus; (**C**) lateral type, rhytids that are limited to the lateral canthus region and (**D**) upper type, rhytid predominantly on the upper side of the lateral canthus.

The pattern of lateral canthal rhytids should be considered before and during the injection. Kane classified lateral canthal rhytids into four types: full, upper, lower and lateral types (Figure 8). The full type has rhytids in the upper, lateral and lower regions. The upper type has rhytids predominantly on the upper side of the lateral canthus. The lower type has rhytids predominantly at the lower side of the lateral canthus, and the lateral type has rhytids that are limited to the lateral canthus region [14]. We propose, in the full type, that three injection points are recommended, while the upper and lower types require two points each, and the lateral type requires one injection point as per proper injection.

Precise injection points and techniques would be related to a lower dose of BoNT. In addition to side effects caused by diffusion at certain points, if increased doses and repeated BoNT injections are administered, antibodies can be produced, which will lead to insufficient treatment [7,8,9,10]. Consequently, broad and thorough anatomical insight into the orbicularis oculi muscle is critical to achieving maximum results with the lowest possible amount of BoNT. If the desired outcomes are not attained, an additional retouching treatment may follow. Likewise, during the injection, manual blocking of the inner boundary of the orbital rim should be carried out [20]. The injection should be performed gently and slowly to prevent diffusion of the BoNT [20].

The limitation of the study is that this proposal has not been compared with the conventional methods.

## 8. Conclusions

This study carried out a broad analysis of published research on the anatomy of the orbicularis oculi muscle in order to provide an anatomical proposal for BoNT indications.

## Data Availability

Not applicable.
